# Using multiple indicators to predict the risk of surgical site infection after ORIF of tibia fractures: a machine learning based study

**DOI:** 10.3389/fcimb.2023.1206393

**Published:** 2023-06-28

**Authors:** Hui Ying, Bo-Wen Guo, Hai-Jian Wu, Rong-Ping Zhu, Wen-Cai Liu, Hong-Fa Zhong

**Affiliations:** ^1^ Department of Emergency Trauma Surgery, Ganzhou People’s Hospital, Ganzhou, China; ^2^ Department of Orthopaedics, Shanghai Jiao Tong University Affiliated Sixth People’s Hospital, Shanghai, China

**Keywords:** machine learning, risk factors, surgical site infection, tibia fractures, predictive model

## Abstract

**Objective:**

Surgical site infection (SSI) are a serious complication that can occur after open reduction and internal fixation (ORIF) of tibial fractures, leading to severe consequences. This study aimed to develop a machine learning (ML)-based predictive model to screen high-risk patients of SSI following ORIF of tibial fractures, thereby aiding in personalized prevention and treatment.

**Methods:**

Patients who underwent ORIF of tibial fractures between January 2018 and October 2022 at the Department of Emergency Trauma Surgery at Ganzhou People’s Hospital were retrospectively included. The demographic characteristics, surgery-related variables and laboratory indicators of patients were collected in the inpatient electronic medical records. Ten different machine learning algorithms were employed to develop the prediction model, and the performance of the models was evaluated to select the best predictive model. Ten-fold cross validation for the training set and ROC curves for the test set were used to evaluate model performance. The decision curve and calibration curve analysis were used to verify the clinical value of the model, and the relative importance of features in the model was analyzed.

**Results:**

A total of 351 patients who underwent ORIF of tibia fractures were included in this study, among whom 51 (14.53%) had SSI and 300 (85.47%) did not. Of the patients with SSI, 15 cases were of deep infection, and 36 cases were of superficial infection. Given the initial parameters, the ET, LR and RF are the top three algorithms with excellent performance. Ten-fold cross-validation on the training set and ROC curves on the test set revealed that the ET model had the best performance, with AUC values of 0.853 and 0.866, respectively. The decision curve analysis and calibration curves also showed that the ET model had the best clinical utility. Finally, the performance of the ET model was further tested, and the relative importance of features in the model was analyzed.

**Conclusion:**

In this study, we constructed a multivariate prediction model for SSI after ORIF of tibial fracture through ML, and the strength of this study was the use of multiple indicators to establish an infection prediction model, which can better reflect the real situation of patients, and the model show great clinical prediction performance.

## Introduction

The incidence of tibia fractures has gradually increased in tandem with the development of the economy and transportation industry. As a serious complication that can occur after open reduction and internal fixation (ORIF) of tibial fractures, surgical site infections (SSI) can lead to serious consequences such as prolonged hospitalization, increased hospital costs, readmissions, osteomyelitis, pseudoarthrosis and even sepsis or death (Henkelmann et al., 2017; Petrosyan et al., 2021; Ying et al., 2021; Barrés-Carsí et al., 2022), which poses a substantial burden to patients and their families. Another noteworthy problem is that the number of patients who develop SSI after discharge from hospital increases with shorter hospitalization times. As preventing SSI is more critical than secondary treatment, clinicians should balance the relationship between infection prevention and shortening hospitalization time. Consequently, it is essential to identify high-risk patients of SSI after tibia fracture and personalize prevention strategies accordingly.

There are many studies that have reported recognized risk factors for SSI, such as diabetes mellitus, obesity, prolonged surgical duration, smoking, elevated inflammatory indicators, etc (Ma et al., 2018; Norris et al., 2019; Ballhause et al., 2021). Additionally, some studies have also highlighted unexpected risk factors such as urinary tract infection (UTI) and bleeding disorders (Yoon and King, 2020; Saiz et al., 2022). To analyze postoperative infections ideally, it is necessary to consider adequate variables. However, most studies’ risk factors are not comprehensive, and simple risk factor analysis has limited clinical application. The use of multiple indicators to develop a prediction model for SSI can be more clinically valuable.

Machine learning (ML) is a form of artificial intelligence that focuses on the use of data and algorithms to predict outcomes, identify patterns and trends within the data and learn from previous experience (Handelman et al., 2018; Liu WC. et al., 2022). ML has demonstrated robust predictive capabilities and is suitable for preoperative medical risk stratification and resource allocation (Ngiam and Khor, 2019; Yeo et al., 2022). In recent years, ML is widely used in the field of medicine, such as for early detection and diagnosis of cancer (Jones et al., 2022), as well as for coronavirus disease 2019 (COVID-19) diagnosis (Pfaff et al., 2022). Although ML has been demonstrated to have greater accuracy than conventional methods, few studies have established ML-based predictive models to identify high-risk patients of SSI, especially in patients with tibia fractures. The purpose of this study was to develop a ML-based predictive model to identify high-risk patients of SSI after ORIF of tibial fractures, which contributes to providing guidance for surgeons to develop personalized prevention and treatment.

## Materials and methods

### Study population

Patients who underwent ORIF of tibial fractures in the Department of Emergency Trauma Surgery at Ganzhou People’s Hospital from January 2018 to October 2022 were retrospectively collected. This study was approved by the Ethics Committee of Ganzhou People’s Hospital. The inclusion criteria were as follows: (1) patients were diagnosed as closed tibial fractures; (2) patients underwent ORIF surgery. The exclusion criteria were as follows: (1) patients with Open injury; (2) patients with multiple site damage; (3) patients with pathological fracture or fracture nonunion; (4) Patients with acute inflammation and infection in other areas of the body; (5) Patients with incomplete data.

### Diagnosis of surgical site infection

The diagnosis of SSI for this study was based on the criteria developed by the Centers for Disease Control in the United States (Horan et al., 1992). In this study, SSI was defined as acute infection within 30 days after ORIF. Patients who met one of the following criteria would be diagnosed as SSI: (1) the wound presented the symptoms or signs of redness, swelling, fever, pain, tenderness to palpation and/or purulent drainage; (2) there was abscess aspirated from the wound and the culture was positive; (3) Fluid or tissue harvested from revision surgery was cultured positively; (4) evidences of SSI was confirmed by histopathologic and radiologic examinations; (5) SSI was diagnosed by the surgeons and definitely noted in the medical records. According to the location of SSI, it was divided into superficial infection and deep infection.

### Data selection

We collected the demographic characteristics, surgery-related variables and laboratory parameters of patients from inpatient electronic medical records, while these variables have been shown to be associated with SSI according to relevant studies (Liu et al., 2018; Norris et al., 2019). Demographic characteristics including Gender, Age, Smoking, Hypertension, Diabetes. Surgery-related variables including Estimated blood loss, Procedure duration, ASA score, Blood transfusion history. Preoperative laboratory parameters including White blood cell (WBC), Neutrophil percentage (%), Lymphocyte percentage (%), Neutrophil count, Lymphocyte count, Red blood cell (RBC), Hemoglobin, Platelet (PLT), Prothrombin time (PT), Activated partial thromboplastin time (APTT), D-Dimer, Total Protein, Albumin, Globulin, Serum glucose, Urinary leukocyte count, Urinary bacterial count. Information on all variables was complete for these patients.

### Statistical analyses

The statistical analyses in this study were all performed by Python (version 3.8, Python Software Foundation). Categorical variables were expressed as frequency or proportions and compared by the chi-square test or Fisher’s exact test. K-S-L test was used to test the normality of continuous data. Continuous non-normally distributed variables were evaluated using the Wilcoxon rank-sum test and shown as median and the first quartile (Q1) and the third quartile (Q3). A significant difference was set as *P*<0.05.

### Data preprocessing, model establishment and performance evaluation

Data of patients were randomly sliced into training and test set in a ratio of 7:3 using a stratified random sampling method in python. Categorical variables such as smoking and diabetes status were processed using label encoding methods. The training set was used to construct the models, and the test set was used to evaluate the prediction performance of models. To address the imbalance of data distribution, random oversampling methods were used. The key of this method is to oversampling the data samples of small classes to increase the number of data samples of small classes to improve the accuracy of the model.

In this study, ten different ML algorithms were constructed with scikit-learn, xgboost and lightgbm modules: Logistic regression (LR), K Neighbors Classifier (KNN), Decision Tree Classifier (DT), Extra Trees Classifier (ET), Random Forest Classifier (RF), Extreme Gradient Boosting (XGBoost), Light Gradient Boosting Machine (Lightgbm), naïve Bayes (NB), Gradient Boosting Classifier (GBC), and Ada Boost Classifier (ADA). The performance of these algorithms was compared without hyper-parameter optimization and the accuracy and area under the receiver operating characteristic curve (AUC) were calculated to select the top three algorithms for further development. The models were then optimized by adjusting the hyper-parameters using the randomized search method, followed by internal and external validation.

Ten-fold cross-validation was used for internal verification, which the training set was split into 10 sets, and nine of them were used for model training and one for model evaluation. The corresponding correct rate was obtained for each trial and the average of the correct rate of the results of 10 times was used as an estimate of the accuracy of the algorithm. AUC and ROC curve were calculated in the test set to externally validate the predictive performance of the ML models. To further evaluate the clinical value of the models, decision curve analyses (DCA) were calculated to show the net benefit of using a model at different thresholds. Calibration was assessed graphically between the predicted and observed outcomes for the training and validation samples. Calibration curve was plotted to assess the calibration of different ML models. Calibration curves depict the calibration of each model in terms of the agreement between the predicted risks of SSI and observed outcomes. Comparing the evaluation indexes of the three model to select the best-performing model.

For the best model, the Youden index, which maximizes the sum of the sensitivity and specificity, was defined to calculate the appropriate cut-off values. External validation was performed using cumulative lift measures to calculate the multiple of the model’s prediction ability compared with the random selection. The confusion matrix intuitively showed prediction performance and the difference between the model prediction result and the real situation. The feature importance and impact of each input variable on the model output was assessed by computing Shapley Additive Explanations (SHAP) values. SHAP was a game-theoretic approach to interpreting the output of ML models. It used the classical Shapley values from game theory and their associated extensions to relate optimal credit allocation to local explanations.

## Results

### Data baseline

According to the inclusion and exclusion criteria, a total of 351 patients underwent ORIF of tibial fractures were included in this study and 242 patients were excluded. Among the patients included, 51 (14.53%) had SSI and 300 (85.47%) without. In 51 patients with SSI, there were 15 cases of deep infection and 36 cases of superficial infection. The detailed characteristics are shown in **Table 1**. The total cohort was split into a training set (n=245) and a test set (n=106) in a ratio of 7:3, while the differences in variables between two groups were not statistically significant (**Table 2**).

**Table 1:** Baseline characteristics of study population.

Variables		Overall	No	Yes	P-Value
**n**		351	300	51	
**Gender, n (%)**	Female	161 (45.9)	139 (46.3)	22 (43.1)	0.672
	Male	190 (54.1)	161 (53.7)	29 (56.9)	
**Age, median [Q1,Q3]**		44.0 [27.5,56.0]	43.0 [27.0,55.0]	50.0 [34.0,56.5]	0.055
**Smoking, n (%)**	No	314 (89.5)	270 (90.0)	44 (86.3)	0.423
	Yes	37 (10.5)	30 (10.0)	7 (13.7)	
**Hypertension, n (%)**	No	285 (81.2)	248 (82.7)	37 (72.5)	0.087
	Yes	66 (18.8)	52 (17.3)	14 (27.5)	
**Diabetes , n (%)**	No	322 (91.7)	285 (95.0)	37 (72.5)	<0.001
	Yes	29 (8.3)	15 (5.0)	14 (27.5)	
**Estimated blood loss, median [Q1,Q3]**		50.0 [30.0,150.0]	50.0 [27.5,100.0]	100.0 [100.0,300.0]	<0.001
**Procedure duration, median [Q1,Q3]**		150.0 [105.0,200.0]	150.0 [99.8,180.0]	220.0 [157.5,292.5]	<0.001
**ASA, n (%)**	1	29 (8.3)	27 (9.0)	2 (3.9)	0.061
	2	311 (88.6)	266 (88.7)	45 (88.2)	
	3	11 (3.1)	7 (2.3)	4 (7.8)	
**Blood transfusion history, n (%)**	No	333 (94.9)	294 (98.0)	39 (76.5)	<0.001
	Yes	18 (5.1)	6 (2.0)	12 (23.5)	
**WBC, median [Q1,Q3]**		9.1 [7.5,11.1]	9.1 [7.4,11.0]	9.8 [8.4,11.6]	0.095
**Neutrophils%, median [Q1,Q3]**		74.5 [68.6,79.8]	73.4 [68.1,79.0]	79.0 [73.3,84.3]	<0.001
**Lymphocyte%, median [Q1,Q3]**		16.9 [12.6,21.7]	17.4 [13.4,22.3]	14.0 [9.3,17.0]	<0.001
**Neutrophil count, median [Q1,Q3]**		6.7 [5.1,8.4]	6.5 [5.1,8.3]	7.5 [6.2,9.2]	0.012
**Lymphocyte count, median [Q1,Q3]**		1.5 [1.2,1.9]	1.5 [1.2,1.9]	1.2 [1.0,1.5]	<0.001
**RBC, median [Q1,Q3]**		4.4 [4.0,4.8]	4.4 [4.0,4.8]	4.2 [3.7,4.6]	0.036
**HB, median [Q1,Q3]**		130.0 [117.0,141.0]	131.0 [118.0,141.0]	129.0 [112.0,138.5]	0.263
**PLT, median [Q1,Q3]**		234.0 [201.0,280.0]	234.0 [202.0,279.2]	234.0 [182.0,282.5]	0.568
**PT, median [Q1,Q3]**		11.2 [10.6,11.8]	11.2 [10.7,11.8]	11.1 [10.4,11.9]	0.631
**APTT, median [Q1,Q3]**		26.2 [24.8,28.3]	26.3 [24.8,28.3]	25.9 [24.4,28.4]	0.619
**D_Dimer, median [Q1,Q3]**		3.9 [1.9,9.4]	3.4 [1.6,8.1]	7.3 [3.5,16.2]	<0.001
**Total Protein, median [Q1,Q3]**		65.9 [63.1,69.7]	65.8 [63.4,69.7]	66.4 [62.0,69.8]	0.364
**Albumin, median [Q1,Q3]**		41.2 [39.2,43.1]	41.4 [39.2,43.2]	40.7 [38.5,42.6]	0.203
**Globulin, median [Q1,Q3]**		24.9 [22.2,27.5]	24.8 [22.2,27.5]	25.1 [22.5,27.4]	0.784
**Glucose, median [Q1,Q3]**		5.5 [5.0,6.2]	5.5 [5.0,6.0]	6.2 [5.5,6.9]	<0.001
**Urinary leukocyte, median [Q1,Q3]**		4.0 [0.0,13.9]	3.7 [0.0,13.7]	5.0 [1.2,19.3]	0.195
**Urinary bacterial, median [Q1,Q3]**		7.2 [0.0,105.5]	7.0 [0.0,95.1]	9.1 [0.0,123.5]	0.471

**Table 2:** Baseline characteristics of training set and test set.

Variables		Overall	Train	Test	P-Value
n		351	245	106	
Infection, n (%)	No	300 (85.5)	209 (85.3)	91 (85.8)	1
	Yes	51 (14.5)	36 (14.7)	15 (14.2)	
Gender, n (%)	Female	161 (45.9)	113 (46.1)	48 (45.3)	0.977
	Male	190 (54.1)	132 (53.9)	58 (54.7)	
Age, median [Q1,Q3]		44.0 [27.5,56.0]	44.0 [27.0,55.0]	46.0 [28.2,57.8]	0.369
Smoking, n (%)	No	314 (89.5)	218 (89.0)	96 (90.6)	0.799
	Yes	37 (10.5)	27 (11.0)	10 (9.4)	
Hypertension, n (%)	No	285 (81.2)	200 (81.6)	85 (80.2)	0.866
	Yes	66 (18.8)	45 (18.4)	21 (19.8)	
Diabetes , n (%)	No	322 (91.7)	228 (93.1)	94 (88.7)	0.247
	Yes	29 (8.3)	17 (6.9)	12 (11.3)	
Estimated blood_loss, median [Q1,Q3]		50.0 [30.0,150.0]	100.0 [30.0,150.0]	50.0 [30.0,150.0]	0.751
Procedure duration, median [Q1,Q3]		150.0 [105.0,200.0]	155.0 [106.0,210.0]	150.0 [96.2,180.0]	0.114
ASA, n (%)	1	29 (8.3)	18 (7.3)	11 (10.4)	0.562
	2	311 (88.6)	220 (89.8)	91 (85.8)	
	3	11 (3.1)	7 (2.9)	4 (3.8)	
Blood transfusion history, n (%)	No	333 (94.9)	232 (94.7)	101 (95.3)	1
	Yes	18 (5.1)	13 (5.3)	5 (4.7)	
WBC, median [Q1,Q3]		9.1 [7.5,11.1]	9.1 [7.5,11.1]	9.1 [7.6,11.0]	0.883
Neutrophils%, median [Q1,Q3]		74.5 [68.6,79.8]	74.1 [68.5,79.3]	74.8 [68.8,81.3]	0.487
Lymphocyte%, median [Q1,Q3]		16.9 [12.7,21.7]	17.0 [13.2,21.9]	16.8 [11.2,21.3]	0.591
Neutrophil count, median [Q1,Q3]		6.7 [5.1,8.4]	6.7 [5.1,8.4]	6.7 [5.3,8.3]	0.809
Lymphocyte count, median [Q1,Q3]		1.5 [1.2,1.9]	1.5 [1.2,1.9]	1.5 [1.1,1.8]	0.716
RBC, median [Q1,Q3]		4.4 [4.0,4.8]	4.4 [4.0,4.8]	4.3 [3.9,4.8]	0.638
HB, median [Q1,Q3]		130.0 [117.0,141.0]	131.0 [117.0,141.0]	129.0 [117.0,140.0]	0.521
PLT, median [Q1,Q3]		234.0 [201.0,280.0]	234.0 [203.0,279.0]	239.5 [193.8,282.8]	0.732
PT, median [Q1,Q3]		11.2 [10.6,11.8]	11.2 [10.6,11.8]	11.2 [10.6,11.8]	0.581
APTT, median [Q1,Q3]		26.2 [24.8,28.3]	26.2 [24.8,28.3]	26.3 [24.7,28.3]	0.76
D_Dimer, median [Q1,Q3]		3.9 [1.9,9.4]	3.9 [1.8,9.5]	3.7 [1.9,8.9]	0.822
Total Protein, median [Q1,Q3]		65.9 [63.1,69.7]	65.9 [63.2,69.8]	66.1 [63.0,69.7]	0.931
Albumin, median [Q1,Q3]		41.2 [39.2,43.1]	41.5 [39.3,43.3]	40.8 [38.8,43.0]	0.173
Globulin, median [Q1,Q3]		24.9 [22.2,27.5]	24.8 [22.2,27.5]	25.2 [22.4,27.7]	0.422
Glucose, median [Q1,Q3]		5.6 [5.0,6.2]	5.5 [5.0,6.1]	5.7 [5.1,6.5]	0.165
Urinary leukocyte, median [Q1,Q3]		4.0 [0.0,13.9]	4.6 [0.4,15.3]	2.3 [0.0,8.9]	0.054
Urinary bacterial, median [Q1,Q3]		7.2 [0.0,105.5]	8.1 [0.0,110.5]	5.4 [0.0,93.6]	0.262

### Candidate algorithms screening

**Figure 1:**
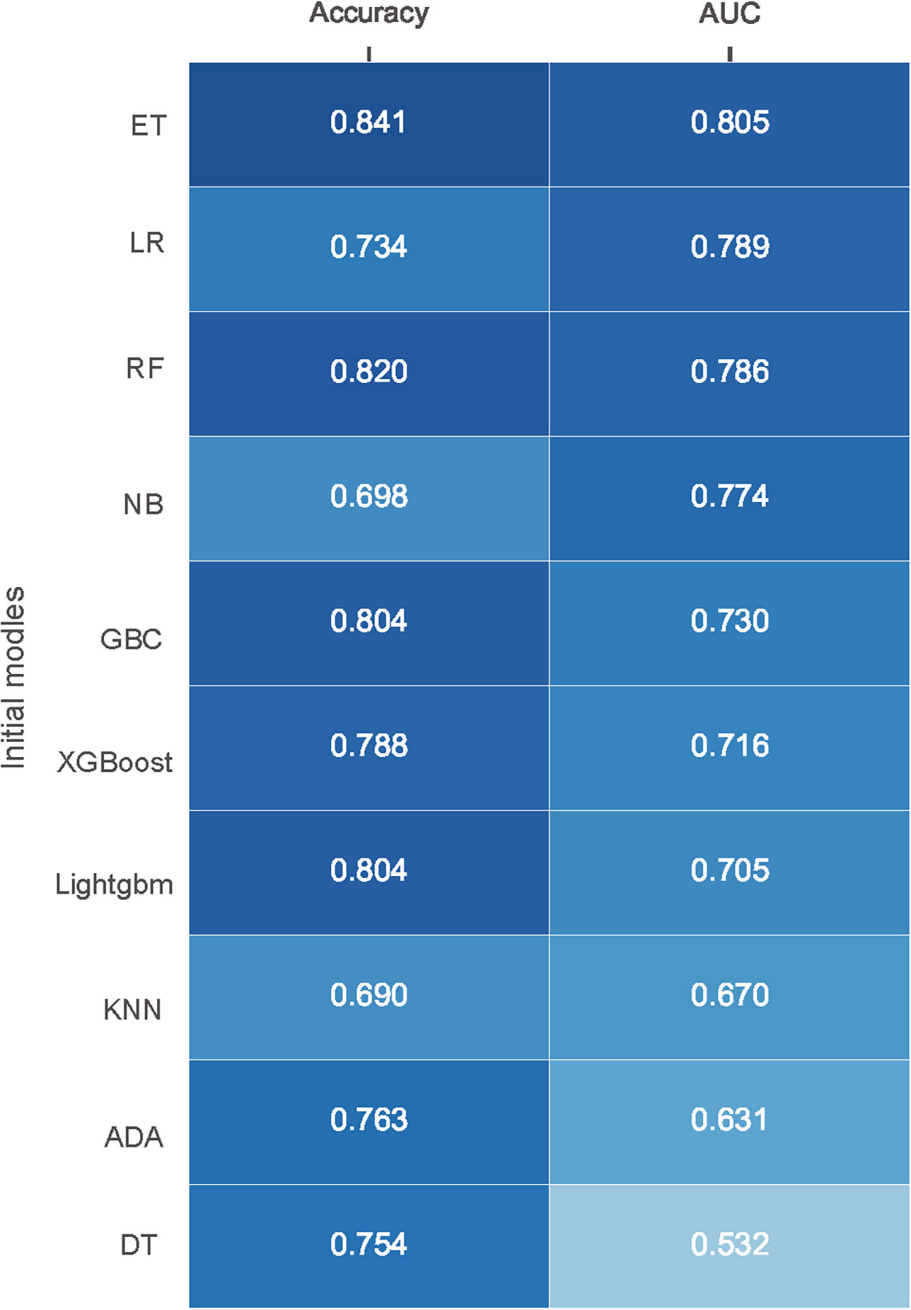
Performance of different models in internal validation without initial parameters. Models are ordered according to their AUC. AUC, area under receiver operating characteristic curve; ET, Extra Trees Classifier; LR, Logistic regression; RF, Random Forest Classifier; NB, Naive Bayes; GBC, Gradient Boosting Classifier; XGBoost, Extreme Gradient Boosting; Lightgbm, Light Gradient Boosting Machine; KNN, K Neighbors Classifier; ADA, Ada Boost Classifier; DT, Decision Tree Classifier.

245 samples were randomly selected for model training. Of these samples, 36 (14.69%) had SSI and 209 (85.31%) without. And, all features were used to construct predictive models in this study. The prediction performances of the various models are exhibited in **Figure 1**. In the initial selection, accuracy and AUC were defined as the main parameters to evaluate the models’ performance. The top three algorithms with excellent performance were selected for the next step experiment, including Extra Trees Classifier (ET) (accuracy: 0.841; AUC: 0.805), Logistic regression (LR) (accuracy: 0.734; AUC: 0.789) and Random Forest Classifier (RF) (accuracy: 0.820; AUC: 0.786).

### Model development and selection

**Figure 2:**
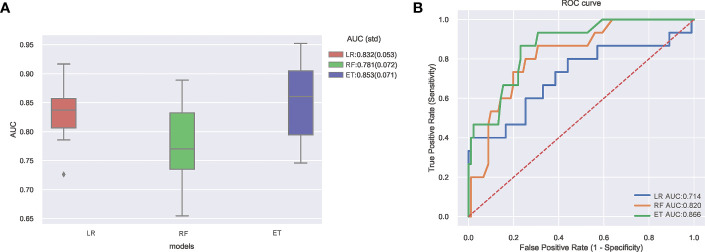
**(A)** Ten-fold cross-validation results of different machine learning models. **(B)** The ROC curves of different machine learning models in external test set. AUC, area under receiver operating characteristic curve; ROC, receiver operating characteristic; ET, Extra Trees Classifier; LR, Logistic regression; RF, Random Forest Classifier.

Internal and external validation after adjusting the optimal hyper-parameter configuration of the models. The final hyperparameters setting of the three models are listed in **Supplementary Table 1**. The performance of the machine learning models was verified by 10-fold cross-validation in the training set, and the results are shown in **Figure 2A**. It can be seen that the ET (AUC: 0.853) model had better performance than LR (AUC: 0.832) and RF (AUC: 0.781) model in internal verification. The ROC curves of three constructed models using the test set are shown in **Figure 2B**. The results also show that the ET model has the best prediction performance with an AUC of 0.866. Both internal validation and external validation show that ET model has the best performance.

### Clinical utility

**Figure 3:**
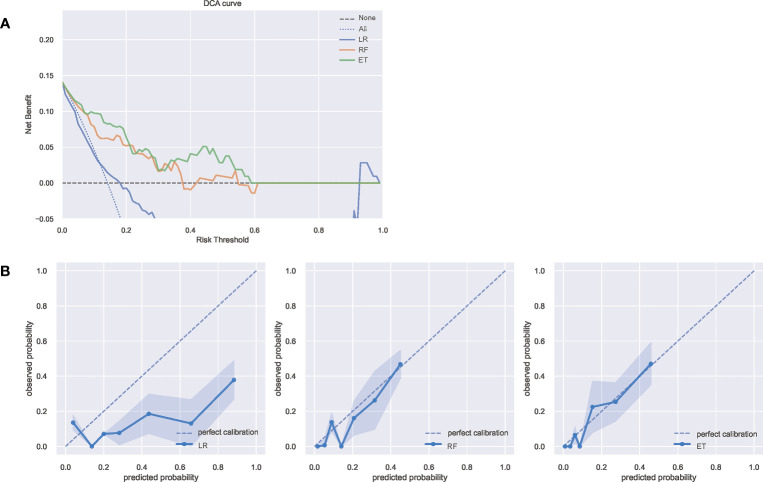
**(A)** The DCA curves of the three model. The net benefit was calculated by adding the true positives and subtracting the false positives. The y-axis represents the net benefit, and the x-axis represents the threshold probability. The Oblique line represents the assumption that all patients will infection, and the horizontal line represents the assumption that no patients with infection. **(B)** The calibration curves of the three models. The y-axis represents the actual infection rate. The diagonal dotted line represents an ideal model and the blue solid line represents the performance of the model, while the model closer fit to the diagonal dotted line represents a better prediction. DCA, decision curve analysis; ET, Extra Trees Classifier; LR, Logistic regression; RF, Random Forest Classifier.

The Decision curve analysis (DCA) of the three model is presented in **Figure 3A**. DCA demonstrated that the ET model added more net benefit compared with RF model or LR model, indicating that it had better clinical impact at a wide range of probability thresholds. Calibration curves depict the calibration of each model in terms of the agreement between the predicted risks of infection and observed outcomes of infection. As shown in **Figure 3B**, the calibration curve of ET model demonstrated good agreement between prediction and observation. The above results show that the ET model has the best clinical performance, so we choose the ET model as the final prediction model to identify high-risk patients of SSI after ORIF of tibial fractures.

### Model performance and feature importance

**Figure 4:**
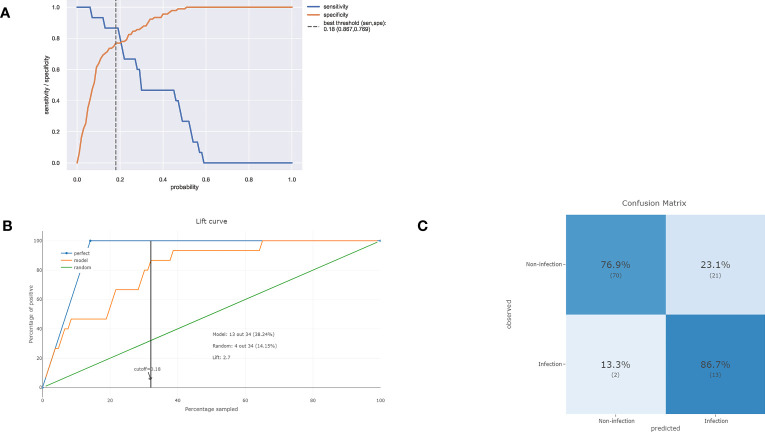
**(A)** Sensitivity and specificity versus cut-off probability plot of the ET model. Decreasing sensitivity and increasing specificity are shown for increasing probability thresholds for infection. **(B)** The cumulative lift demonstrates a snapshot of the ratio of the percentage of patients with infection events reached during a treatment campaign to the percentage of patients targeted. It showed the rate of positive events captured by a model over a given number of samples. **(C)** The confusion matrix of the ET model in the test set.

**Figure 5:**
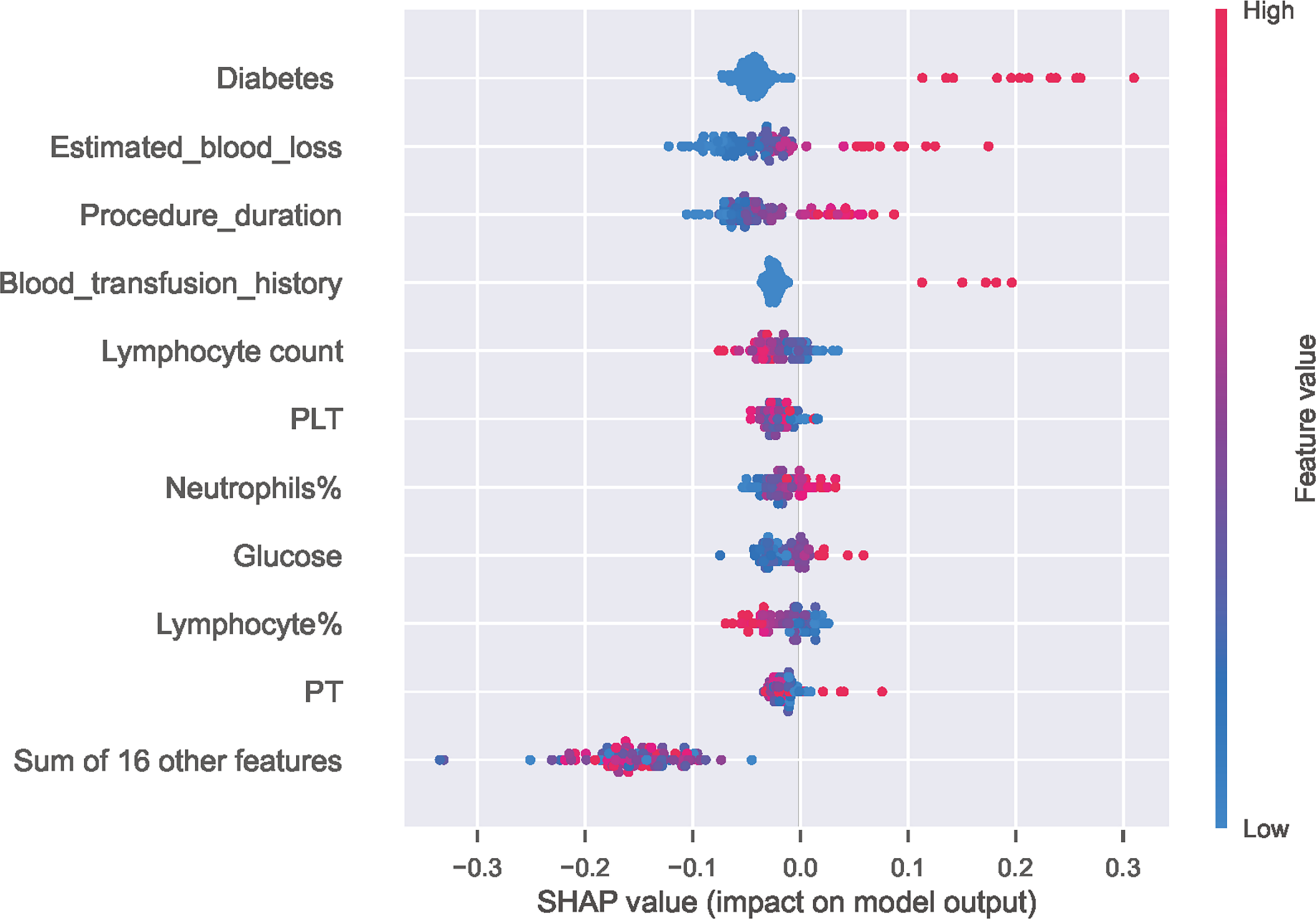
Distribution of the impact of each feature on the output of ET model estimated using the SHAP values. The plot sorts features by the sum of SHAP value magnitudes over all samples and shows the order of feature importance. This figure described data from the test cohort, with each point representing one patient. The color represents the feature value (red high, blue low). The x axis measures the impact on the model output (right positive, left negative). A positive value indicate a SSI risk and a negative value indicate a good outcome. SHAP, SHapley Additive explanation; PLT, Platelet; PT, Prothrombin time.

As illustrated in **Figure 4A**, decreasing sensitivity and increasing specificity are shown for an increasing probability of infection, with a histogram for the distribution of the predicted probability. We defined an optimal cut-off probability of 0.18 for the ET model according to the Youden index and the sensitivity and specificity were 0.867, 0.769 respectively. **Figure 4B** demonstrates the cumulative gains of ET model, which showed the rate of SSI events captured by ET model over a given number of samples. The cumulative lift demonstrates a snapshot of the ratio of the percentage of patients with infection to the percentage of patients without. We used it to compare ET model vs a theoretically ideal model (perfectly predicts SSI given a sample) and a model that is no better than random guessing. When cut-off value was 0.18, the lift value was 2.7 for the ET model. The confusion matrix (**Figure 4C**) of the ET model in the test set indicated its great prediction performance. To reveal the relative importance of features in ET model, SHAP values were calculated and are plotted in **Figure 5**. As shown, Diabetes, Estimated blood loss, Procedure duration, Blood transfusion history are the most important features for distinguishing the SSI and non-SSI groups.

## Discussion

Tibial fractures are a frequent and complicated injury for orthopedic surgeons, typically resulting from high-energy trauma and often leading to complications (Choo and Morshed, 2014; Henkelmann et al., 2017). Identification high-risk patients for surgical site infection (SSI) and providing personalized prevention and treatment are important considerations. This study included 351 patients, of whom 51 developed postoperative infections. Previous literature reported a wide range of the rate of SSI between 2.6–45% for tibial fractures (Henkelmann et al., 2017), while our results show that the overall incidence of SSI is 15%, and 4.1% for deep SSI, falling within the previously reported range. Compared to other types of fractures, there is a higher incidence rate of SSI after ORIF of tibial fractures (Shen et al., 2021), and the possible cause for this difference is that tibia is covered with sparse soft tissue and usually suffers from severe injuries (Norris et al., 2019).

ML has been widely used in the medical field, and a ML-based multivariate prediction model was constructed to screen out high-risk patients of SSI in this study. Ten algorithms including LR, KNN, DT, ET, RF, XGBoost, Lightgbm, NB, GBC and ADA were used to predict SSI risk after ORIF of tibial fractures. Through internal and external validation, it is found that ET model has the best prediction performance, and the test set AUC of ET model is 0.896, moreover, the prediction model also shows great clinical performance.

In the models we constructed, all the included indicators are the possible risk factors for SSI according to previous literature (Liu et al., 2018; Norris et al., 2019) and these indicators mainly fall into three categories: demographic characteristics, surgery-related variables and laboratory parameters. Predictor variables should be included as much as possible to better reflect the actual situation of patients, but some risk factors for SSI are excluded because they cannot be collected or difficult to measure, for instance, it is difficult to weigh fracture patients, so BMI cannot be calculated, and wound dressing is greatly affected by the experience and habits of the clinician and cannot be measured. To our knowledge, this is the first to include almost all collectable indicators to develop a ML-based prediction model for SSI after ORIF of tibia fractures. Through the feature importance experiment, we identified several indicators that have the greatest impact on the model, and diabetes, estimated blood loss, procedure duration, blood transfusion history, lymphocyte count and PLT are the top six.

Our results show that the diabetes was the most important variable in the model, and lots of research have indicated diabetes is an independent risk factor for SSI. Such as, Bachoura et al. found that diabetes is the nonmodifiable risk factors for SSIs after skeletal trauma (Bachoura et al., 2011), and Oladeji et al. show that diabetic patients were 2.7 times more likely to develop a deep infection than ordinary patients after pilon fracture fixation (Oladeji et al., 2021). The microvascular blood flow of patients with diabetes is usually damaged, which decreased the ability to deliver antibiotics or inflammatory cells to the injured area to resist infection, resulting in the higher risk of SSI. Diabetic status was identified by the medical records, and we noticed that glucose control in patients with diabetes was different, which may have different effects on the occurrence of infection. Similar study has been reported by Anderson et al. that glucose ≥200 mg/dL was a significant independent risk factor for 90-day deep surgical site infections in orthopaedic trauma patients (Anderson et al., 2021). In addition, Andres et al. indicated that stress-induced hyperglycemia also increased the risk of infection in orthopedic trauma patients without a history of diabetes (Rodriguez-Buitrago et al., 2019). Therefore, diabetes alone cannot well reflect its impact on infection, while the combination of serum glucose and diabetes can produce a better predictive value.

Prolonged operative time is a well-accepted risk factor for SSI after tibia fractures. A retrospective analysis of 309 tibial plateau fractures found operative times approaching 3 hours was related to an increased risk for SSI (Colman et al., 2013). Li et al. also showed that patients who developed SSI after surgery had a longer operative time (200.5 ± 82.5 min) than those without infection (142.8 ± 54.1 minutes) (Li et al., 2018). Prolonged operative time not only results in more extensive soft-tissue stripping and extended exposure of the wound but also leads to higher estimated blood loss (EBL). EBL, as an independent predictor of SSI after orthopedic surgery, has been widely reported in the previous literature (Li et al., 2015; Liu WC. et al., 2022), and our results also show that the EBL plays an important role in infection prediction. Moreover, our study found that patients with SSIs had a higher proportion of blood transfusion history compared with patients without SSIs. Similar evidence was also found in Panteli et al.’s study, and it show post-operative transfusion associations with deep infection (Panteli et al., 2021). Additionally, some studies believe that Immunosuppression as the consequence of blood transfusion is related to the increase of SSI rate (Hill et al., 2003; Guerado et al., 2016). From a clinical perspective, patients who need blood transfusion after surgery are associated with more blood loss or poor physical condition, and all these factors may increase the rate of SSI. Operation time, EBL, and blood transfusion history influence each other to varying degrees, and they should not be considered in isolation.

In contrast to patient and intraoperative factors, which are sometimes subjective and unclear in showing body status, serum biomarkers are more sensitive and objective (Wu et al., 2022). The abnormal preoperative inflammatory indicators is not only the reflection of the acute inflammatory response but also the stress response of body to injury, which may contribute to the prediction of infection. Zhao et al. reported that the increase in preoperative inflammatory markers such as WBC count is significantly associated with SSI (Zhao et al., 2022), and Lu et al. showed that NLR (the values of ratio of neutrophil to lymphocyte) ≥6.4 is independently associated with SSI (Lu et al., 2022). Some scholars believed that lymphocytes represent the immune function of patients and thus are associated with infection (Iwata et al., 2016). Consistent with the previous literature, we also found that some inflammatory indicators had statistically significant differences between SSI and non-SSI patients and played an important role in model construction in this study, such as neutrophil and lymphocyte. As Imabayashi et al. found that the combination of neutrophil count, lymphocyte ratio, and C-reactive protein ratio, may be a strong tool for detecting SSI (Imabayashi et al., 2022), Thus, it may be a better choice to use a combination of inflammation indicators to predict infection.

Although the univariate analysis did not suggest a significant correlation between the PLT and SSI, we cannot completely ignore its importance in infection prediction. Liu et al. reported that the temporal changes of the PLT count in immunocompromised patients who have undergone femoral neck fracture repair can serve as an early warning of SSI (Liu J. et al., 2022). Zhang et al. posit that platelet count were significantly higher in the Deep surgical site infection (DSSI) group than in the non-DSSI group PLT after ORIF for traumatic limb fractures (Zhang et al., 2018). On the other hand, Hu et al.’s study found that PLT<288 × 10^9^ is an independent risk factor for wound infection after surgical treatment of open fractures (Hu et al., 2020). Saiz et al. found that patients with bleeding disorder are more likely to develop SSI than patients without (Saiz et al., 2022). The lack of statistical difference in PLT indicators may be due to the limited sample size in our study. However, it is important to note that PLT has a significant impact on the ML-based predictive model for SSI, which highlights the potential benefits of using ML methods to detect subtle associations that may not be apparent with traditional statistical approaches.

It is worth noting that the prediction model in this study was established for clinical purposes, and the ET model demonstrated good clinical performance. The confusion matrix showed that 13 out of the 15 infected patients were correctly predicted in the external test. Further prospective clinical prediction tests are needed to verify the actual effectiveness of the model. According to the risk assessment results generated by the prediction model, clinicians should pay more attention to high-risk patients of SSI and develop personalized treatment. Furthermore, this screening approach help to strike a balance between shortening the average hospital stay and minimizing post-discharge infection rates; screening high-risk patients allows for selective extension of hospitalization for those at high risk, while potentially reducing the hospitalization duration for low-risk patients.

## Limitations

This study has several limitations. First, this is a retrospective study, which limits the source of data to medical records and reduces the credibility of the evidence. Additionally, the retrospective study design may introduce selection bias, and patients with confirmed SSI in other institutions were excluded. Furthermore, the number of cases in this study was relatively small, and it was a single-center research. Future studies should be conducted in multiple centers with larger sample sizes.

## Conclusions

In summary, this study constructed a multivariate prediction model for SSI after ORIF of tibial fracture using ML. The use of multiple indicators to establish the infection prediction model was a significant strength of this study, which can better reflect the real situation of patients. The model demonstrated good clinical prediction performance, which contributes to the screening and personalized treatment of high-risk patients of SSI.

## Data availability statement

The raw data supporting the conclusions of this article will be made available by the authors, without undue reservation.

## Ethics statement

The studies involving human participants were reviewed and approved by the Ethics Committee of Ganzhou People’s Hospital. The patients/participants provided their written informed consent to participate in this study.

## Author contributions

HY, W-CL and H-FZ conceived of and designed the study. HY, W-CL, B-WG, H-JW and R-PZ performed analysis and generated the figures and tables. HY and W-CL wrote the manuscript and HY, W-CL and H-FZ critically reviewed the manuscript. All authors have read and approved the manuscript.
