# Supplementary material for: Using multiple indicators to predict the risk of surgical site infection after ORIF of tibia fractures: a machine learning based study
Source: Front Cell Infect Microbiol. 2023 Jun 28;13:1206393. doi: 10.3389/fcimb.2023.1206393 (PMC10338008; doi:10.3389/fcimb.2023.1206393)
Supplement: Supplementary file 1 [file Table_1.docx]

| **Supplementary Table 3.** Final hyperparameters setting of the three models. |
| --- |
| Extra Trees Classifier (ET)  Extra Trees Classifier (bootstrap=True, ccp_alpha=0.0, class_weight='balanced',  criterion='entropy', max_depth=7, max_features='sqrt',  max_leaf_nodes=None, max_samples=None,  min_impurity_decrease=0.05, min_samples_leaf=4,  min_samples_split=9, min_weight_fraction_leaf=0.0,  n_estimators=230, n_jobs=-1, oob_score=False,  random_state=123, verbose=0, warm_start=False) |
| Logistic Regression (LR)  Logistic Regression (C=0.056, class_weight={}, dual=False, fit_intercept=True,  intercept_scaling=1, l1_ratio=None, max_iter=1000,  multi_class='auto', n_jobs=None, penalty='l2',  random_state=123, solver='lbfgs', tol=0.0001, verbose=0,  warm_start=False) |
| Random Forest Classifier (RF)  Random Forest Classifier (bootstrap=True, ccp_alpha=0.0, class_weight=None,  criterion='gini', max_depth=None, max_features='sqrt',  max_leaf_nodes=None, max_samples=None,  min_impurity_decrease=0.0, min_samples_leaf=1,  min_samples_split=2, min_weight_fraction_leaf=0.0,  n_estimators=100, n_jobs=-1, oob_score=False,  random_state=123, verbose=0, warm_start=False) |
